# Identification and Verification of a Prognostic Risk Signature in Oral Squamous Cell Carcinoma

**DOI:** 10.2174/0115680266335055240828061128

**Published:** 2024-09-05

**Authors:** Rishou Chen, Junlin Duan, Yonglong Ye, Huan Xu, Yali Ding, Jun Liu

**Affiliations:** 1 Laboratory Medicine, Dongguan Hospital of Guangzhou University of Chinese Medicine, Dongguan, China

**Keywords:** OSCC, mTORC1, ssGSEA, Gene mutation, Risk signature, WGCNA, TCGA

## Abstract

**Introduction:**

Oral squamous cell carcinoma (OSCC) is a prevalent malignant condition. This study aimed to investigate the role of mTORC1 signaling and develop a prognostic model for OSCC.

**Materials and Methods:**

The single-sample gene set enrichment analysis (ssGSEA) algorithm was utilized to calculate the Z-Score of Hallmarks in OSCC, followed by univariate Cox regression analysis to identify processes associated with prognosis. Weighted gene co-expression network analysis (WGCNA) was performed using transcriptomic data from the cancer genome atlas (TCGA) cohort to identify genes correlated with mTORC1 signaling. A six-gene prognostic model was constructed using multifactorial Cox regression analysis and validated using an external dataset.

**Results:**

The study uncovered a strong linkage between mTORC1, glycolysis, hypoxia, and the prognosis of OSCC. mTORC1 signaling emerged as the most significant risk factor, negatively impacting patient survival. Additionally, a six-gene prognostic risk score model was developed which provided a quantitative measure of patients' survival probabilities. Interestingly, within the context of these findings, TP53 gene mutations were predominantly observed in the high-risk group, potentially underlining the genetic complexity of this patient subgroup. Additionally, differential immune cell infiltration and an integrated nomogram were also reported.

**Conclusion:**

This study highlights the importance of mTORC1 signaling in OSCC prognosis and presents a robust prognostic model for predicting patient outcomes.

## 1 INTRODUCTION

Oral squamous cell carcinoma (OSCC) is an invasive neoplasm that arises from the epithelial cells of the oral mucosa [1]. Common clinical manifestations include maxillofacial ulcers, leukoplakia, erythema, and palpable masses, leading to significant impairment in oral function and overall quality of life for affected individuals. On a global scale, oral cancer ranks as the sixth most prevalent malignancy [2]. The World Health Organization reports an annual incidence of over 300,000 oral cancer cases worldwide, with a disproportionate burden of 62% occurring in developing nations, particularly within the Asia [3]. The delayed detection of OSCC presents a significant prognostic obstacle for individuals [4]. Despite the availability of various treatment options, such as chemoradiotherapy, surgical resection, and immunotherapy, the five-year survival rate remains below 50 percent. Consequently, it is imperative to discover novel biomarkers, establish new prognostic models, categorize patients based on risk, and refine their management. Additionally, personalized treatment decisions and monitoring protocols should be implemented to optimize treatment strategies and enhance the prognosis and survival outcomes for individuals with OSCC.

In recent years, bioinformatics has become an important field, combining molecular biology and information technology [5]. Bioinformatics employs sophisticated computational techniques and leverages biological databases to process and analyze biological data. This allows for comprehensive investigations into the complex molecular mechanisms underlying diseases. Bioinformatics plays a crucial role, particularly in tumor research. Through the meticulous analysis of diverse histological data encompassing gene expression, mutations, protein levels, and more, bioinformatics can discern pivotal molecules intricately linked to the progression of tumors. These molecules may encompass novel tumor markers or disease-associated genes, which play a crucial role in elucidating tumor pathogenesis. Notably, recent studies have provided additional evidence showcasing the utility of bioinformatics in tumor research. For instance, Chi *et al*. devised a prognostic model utilizing head and neck squamous cell carcinoma data from The Cancer Genome Atlas (TCGA) database to predict clinical outcomes in head and neck cancer. This model relies on the analysis of 17 genes linked to NK cells and demonstrates its ability to forecast patient prognosis [6]. This model holds the potential to aid physicians in assessing patient risk levels and strategize appropriate treatments. Similarly, Liu *et al*. utilized TCGA data on hepatocellular carcinoma to construct an innovative predictive model that incorporated six genes. These were identified as independent risk factors for hepatocellular carcinoma. The model exhibited proficient prognostic capabilities for patients with hepatocellular carcinoma [7]. By utilizing such a model, it is plausible to enhance the precision of prognosis assessment for individuals with liver cancer and potentially devise tailored treatment plans for them in forthcoming endeavors. In addition, Li *et al*. employed single nucleotide variant and transcriptome data from TCGA to investigate the aberrant expression of m6A regulators in colorectal cancer [8]. The development of prognostic signatures using m6A-associated long non-coding RNAs (lncRNAs) and messenger RNAs (mRNAs) independently was pursued. Notably, both models demonstrated strong predictive capacities, providing invaluable insights into prognostic risk assessment for colorectal cancer patients and supporting informed therapeutic decisions. Through the use of bioinformatics to draw out vital molecular characteristics from comprehensive tumor data, these studies make significant contributions to clinical practice by creating predictive models. These models assist physicians in prognostic evaluations and risk assessments while revealing key pathways and biological processes involved in tumor development. Moreover, performing thorough functional enrichment analysis can further our understanding of the irregular enrichment of oncological features and metabolic processes in high-risk groups. Such studies significantly contribute to advancing precision medicine and personalized therapy. However, more research is required to discover prognostic markers for OSCC.

In this study, we used OSCC expression profiles from the TCGA database and the single-sample gene set enrichment analysis (ssGSEA) algorithm to evaluate the predictive value of genes associated with OSCC hallmarks. We then used WGCNA and other methods to identify key genes linked to OSCC prognosis. Subsequently, we crafted a 6-gene prediction model by coupling machine learning with Cox regression. In order to ascertain the dependability of the model, the research team employed an independent dataset sourced from the Gene Expression Omnibus (GEO) database for external validation. This study also explored the link between prognostic risk scores, immune infiltration, and tumor mutation burden. The findings provide new insights for improving OSCC patient care and prevention, supporting personalized treatment, and advancing management strategies.

## 2 MATERIALS AND METHODS

### 2.1 Data Collection and Processing

The RNA sequencing (RNA-seq) data of head and neck squamous cell carcinoma (HNSC) were obtained from the TCGA (The Cancer Genome Atlas, https://portal.gdc.cancer.gov/) database for this study. Expression profiles from various tissue sites, including alveolar ridge, buccal mucosa, floor of mouth, tongue, lips, oral cavity, and hard palate, were screened. A total of 32 normal samples and 319 tumor samples from OSCC were acquired. To facilitate subsequent analyses, the data were converted to transcripts per million mapped reads (TPM) format and normalized. Additionally, we acquired the tumor mutation load data along with the corresponding clinical staging and follow-up data, which offer valuable insights into the mutational status and disease staging of the patients. Furthermore, we accessed the expression profiling data of 97 cases of OSCC from the Gene Expression Omnibus (GEO, https://www.ncbi.nlm.nih.gov/geo/) database (GSE41613) and performed normalization. Additionally, we collected the corresponding clinical follow-up data for these cases.

### 2.2 Identification of Potential Biomarkers

In this study, we utilized the GSEA database (https://www.gsea-msigdb.org) to obtain a list of “hallmark genesets.” These gene sets represent specific biological processes or functional modules. To determine the enrichment score of each gene set in the samples, we applied the ssGSEA algorithm. This algorithm maps the gene expression data to the defined gene sets and calculates the enrichment score for each gene set in the sample. By using ssGSEA, we can assess the enrichment level of each “hallmark gene set” in the samples, revealing the relevance of these gene sets to the biological processes and functional modules represented by them. To make the enrichment scores of different gene sets comparable and reflect the relative enrichment in the samples, we normalized the enrichment scores of all gene sets, resulting in the final ssGSEA score. Subsequently, we evaluated the prognostic significance of each Hallmark using a univariate Cox proportional hazards (Cox-PH) regression model. The Cox-PH model is a commonly used statistical model in survival analyses, allowing us to assess the relationship between genes or gene sets and survival time. To identify modules associated with the mTORC1 signature, we constructed an unscaled signed co-expression network based on weighted gene co-expression network analysis (WGCNA). Next, we conducted a univariate Cox regression analysis to filter genes from mTORC1 signature-related modules that demonstrated prognostic significance. By calculating hazard ratios (risk ratios) and confidence intervals, we determined the importance of genes in prognosis. The Human Protein Atlas (HPA) is a globally recognized database that serves as a comprehensive resource for human protein information. Utilizing the HPA database, we conducted validation of the protein expression levels for our identified potential biomarkers in both normal and tumorous tissue samples.

### 2.3 Construction of a Prognostic Risk Signature

**Fig. (1):**
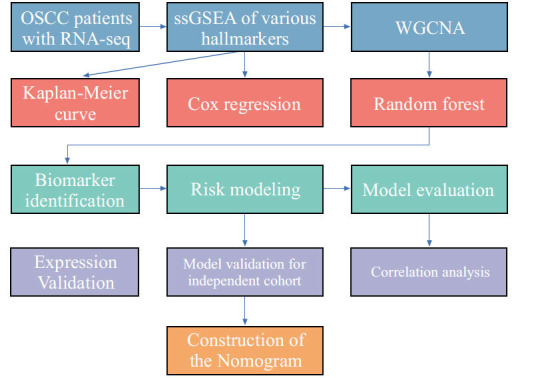
The flowchart of this study.

Random Forest algorithm was employed to evaluate the relative importance of prognostic genes in predicting outcomes. Random Forest is a powerful machine learning algorithm that generates multiple decision trees and combines their results to assess the importance of variables. The Random Forest algorithm assesses the significance of individual genes in predicting the prognosis of OSCC by evaluating their impact in the construction of decision trees, subsequently identifying the top 10 ranked genes as principal features. Finally, we performed multifactorial Cox regression analysis by randomly combining these ten genes, resulting in 1023 prognostic models (210 - 1). To assess the prognostic value of the developed model, we calculated risk scores for each patient based on the prognostic model. Using the median value as the threshold, we divided OSCC patients into high-risk and low-risk groups. We then employed the Kaplan-Meier (K-M) method to compare the overall survival time between these two risk groups. This analysis allowed us to evaluate the association between the risk score and patient outcomes. To further evaluate the predictive accuracy of the risk model, we utilized receiver operating characteristic (ROC) curves. ROC curves provide a graphical representation of the trade-off between sensitivity and specificity for different risk score thresholds. By calculating the area under the curve (AUC), we assessed the predictive accuracy of the risk model. A higher AUC indicates better predictive performance. Furthermore, to validate the prognostic impact and predictive accuracy of our model, we conducted an independent validation using the GSE41613 dataset (97 OSCC cases). This external dataset served to verify the robustness and generalizability of our findings. By applying our model to this validation set, we could assess its performance in an independent cohort, strengthening the reliability of our results (Fig. **1**).

### 2.4 Correlation Analysis Between Risk Score and Immune Microenvironment

To investigate the relationship between the risk score and the immune microenvironment, we conducted ssGSEA analysis to obtain the levels of immune infiltration for various immune cell types in both the high-risk and low-risk groups. By visually comparing these results, we can present them in a bar graph format. This graphical representation allows for a clear comparison of the relative levels of different immune cells between the two groups. It provides a quick overview of the differences in immune cell infiltration. Furthermore, to enhance our understanding of the immune microenvironment, we can include the immune infiltration levels of immunosuppressive gene checkpoints in the same bar graph. These gene checkpoints are known to regulate and restrict immune responses, potentially playing a pivotal role in the tumor microenvironment. By comparing the levels of immune infiltration for these gene checkpoints between the high-risk and low-risk groups, we can assess the relationship between the risk score and the mechanism of immunosuppression. This analysis will provide insights into how the risk model is associated with immunosuppressive processes within the tumor microenvironment.

### 2.5 Bioinformatics and Statistical Analysis

This study utilized R software (version 4.1.1) for data analysis and graphical visualization. The ssGSEA scores and genetic trait-based risk scores were normalized using the Z-score method to ensure comparability between different scores and to facilitate a more precise assessment of their impact on the results. Survival curves were plotted using the Kaplan-Meier method to compare differences in survival between high-risk and low-risk groups. A log-rank test allowed us to quantitatively assess statistical differences between these groups. The Cox-PH regression model was used to assess the significance of each parameter on overall survival (OS). This model facilitates the examination of relationships among multiple variables and assesses the independent impact of each variable on survival outcomes. The predictive efficacy of the risk model was evaluated using time-dependent Receiver Operating Characteristic (t-ROC) analysis. The model's accuracy and sensitivity were quantified through the calculation of the AUC. To compare the risk scores between the two patient cohorts and determine the statistical significance of differences between the high-risk and low-risk groups, the “Wilcox. test” function was employed (supplementary figures).

## 3 RESULTS

### 3.1 MTORC1 Signaling is a Critical risk Factor for OSCC

**Fig. (2):**
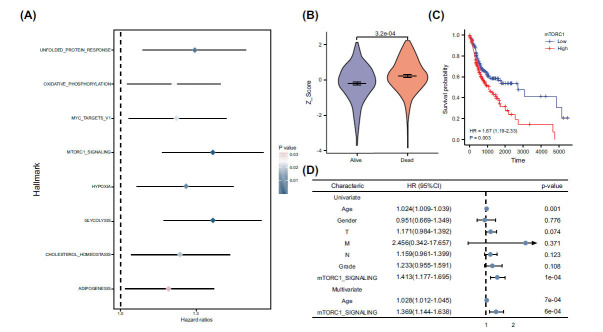
Identification of mTORC1 signaling as a critical risk factor in Oral Squamous Cell Carcinoma (OSCC). (**A**) Employing Single-Sample Gene Set Enrichment Analysis (ssGSEA) and univariate Cox regression algorithms, we evaluated the prognostic significance of various hallmarkers across OSCC patients. (**B**) The Z-score for mTORC1 signaling was significantly elevated in OSCC patients who succumbed to the disease compared to those who survived, indicating its potential role in determining patient outcomes. (**C**) Kaplan-Meier analyses illustrated that patients with high mTORC1 signaling Z-scores had a notably reduced overall survival period relative to their counterparts with low Z-scores, thereby underscoring the influence of mTORC1 signaling levels on survival rates. (**D**) Both univariate and multifactorial Cox regression analyses consistently highlighted mTORC1 signaling as an independent prognostic factor for OSCC, further reinforcing its importance in prognosis prediction. A Mann-Whitney U test (Wilcoxon rank sum test) was used to determine the P values.

We utilized the ssGSEA (single-sample gene set enrichment analysis) algorithm to calculate the Z-Score of Hallmarks in OSCC. Through subsequent univariate Cox regression analysis, several processes, including cholesterol homeostasis, glycolysis, adipogenesis, hypoxia, and Myc targets, were identified as significant contributors to the prognosis of OSCC. The flowchart illustrates the overall design of the study (Fig. **1**). This suggests a close association between these processes and the development and prognosis of OSCC. Our results revealed that the mTORC1 signaling pathway was the most significant risk factor, with higher Z-scores observed in patients who succumbed to the disease (Figs. **2A**, **2B**). Additionally, Kaplan-Meier survival analysis demonstrated a lower overall survival rate in the high Z-Score group associated with mTORC1 signaling compared to the low Z-Score group among OSCC patients (Fig. **2C**). Furthermore, multifactorial COX analysis highlighted mTORC1 as an independent prognostic factor for overall survival in OSCC patients (Fig. **2D**). These findings strongly suggest that activation of the mTORC1 signaling pathway may be closely associated with poor prognosis in OSCC patients.

### 3.2 Construction of mTORC1 Signaling-related Prognostic Model

**Fig. (3):**
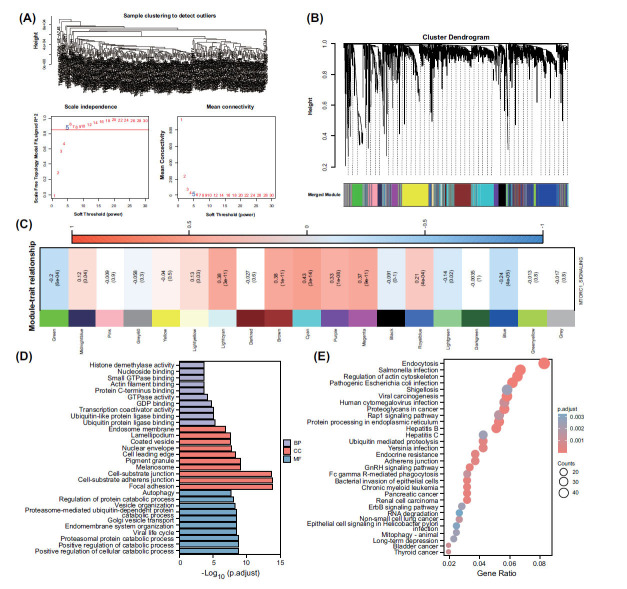
Identification and functional analysis of potential biomarkers related to mTORC1 signaling. (**A**) Assessing the weighted gene co-expression network analysis (WGCNA) model fit and determining the optimal soft threshold. (**B**) In the WGCNA analysis, a soft threshold of 5 was determined as the final value for subsequent analysis. Using this soft threshold, a total of 19 gene co-expression modules were constructed. (**C**) The correlation and p-values of the 19 gene co-expression modules with mTORC1 signaling were assessed to determine their association with this pathway. (**D**) The Gene Ontology (GO) functional analysis of genes within the cyan, lightcyan, brown, purple, and magenta modules. (**E**) The Kyoto Encyclopedia of Genes and Genomes (KEGG) functional analysis of genes within the cyan, lightcyan, brown, purple, and magenta modules.

To construct a prognostic model related to mTORC1 signaling, we initially screened 6443 genes using ANOVA based on their variance values (>1). These genes exhibited high variability and are likely to play important biological functions in OSCC. Subsequently, we carried out a WGCNA on the TCGA cohort's transcriptomic data for the identified 6443 genes. Choosing β=5 as the soft threshold facilitated measuring gene co-expression correlations in the signed weighted network (Fig. **3A**), hence enabling gene classification into distinct modules. The application of this threshold resulted in 19 co-expression modules (Fig. **3B**). These modules were formed based on similarities between genes and provided insights into the role of gene regulatory networks and pathways in OSCC. Among these modules, the cyan, light cyan, brown, purple, and magenta modules (comprising a total of 2481 genes) exhibited a high co-expression correlation with mTORC1 (r>0.3, p<0.001) (Fig. **3C**). This indicates that the genes within these modules may be closely associated with mTORC1 in OSCC and may participate in common biological processes or signaling pathways. To elucidate the potential functions of these genes, we performed enrichment analyses using Gene Ontology (GO) and the Kyoto Encyclopedia of Genes and Genomes (KEGG) databases. The GO analysis indicated that these genes may be involved in autophagy, regulation of protein catabolic process, positive regulation of cellular catabolic process, and other processes (Fig. **3D**). Additionally, the KEGG analysis suggested their potential association with endocytosis, salmonella infection, hepatitis B, adherens junction, and other pathways (Fig. **3E**).

**Fig. (4):**
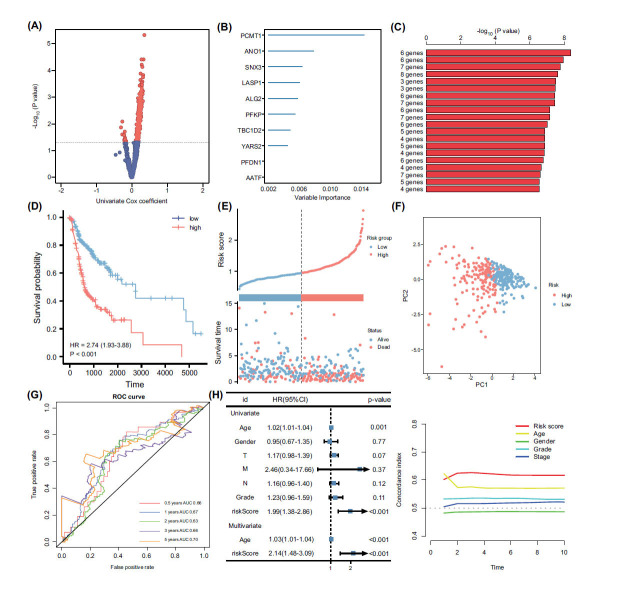
Identification of key prognostic-related genes and construction of a prognostic risk signature. (**A**) A total of 554 genes were identified as being prognostically relevant through univariate COX regression analysis. (**B**) The random forest importance measure to rank genes based on their prognostic importance for oral squamous cell carcinoma (OSCC). This approach allowed us to identify the top 10 genes that exhibited the highest significance in predicting OSCC prognosis. (**C**) By conducting a Kaplan-Meier analysis, we assessed the relationship between the expression levels of these gene signatures and patient survival outcomes. The -log10 p-value served as an indicator of the statistical significance of the association between each gene signature and OSCC prognosis. (**D**) The Kaplan-Meier method was employed to generate survival curves based on the risk scores calculated from our prognostic model. (**E**) The distribution of risk scores and overall survival status was examined for the high- and low-risk groups in the training cohort. (**F**) The principal component analysis (PCA) was conducted to assess the differentiation between the high and low-risk groups in training cohort. (**G**) The receiver operating characteristic (ROC) curve was utilized to evaluate the predictive efficacy of the risk model in the training cohort. (**H**) Univariate and multivariate COX regression demonstrated this risk model to be an independent prognostic factor. (**I**) Concordance index was utilized to analyze the predictive efficacy of our risk model in comparison to different clinical indicators for prognosis assessment.

To further identify genes associated with OSCC prognosis, the univariate Cox analysis was conducted on the 2481 genes found to exhibit significant co-expression correlation with mTORC1. The analysis revealed that 554 genes displayed a significant association with oral cancer prognosis (Fig. **4A**). These genes likely play a crucial role in prognostic assessment and predicting patient outcomes. Subsequently, a random forest algorithm was employed to rank the effects of these 554 genes on prognosis, leading to the selection of the top ten ranked genes as candidate genes (Fig. **4B**). These top-ranked genes were considered to have a substantial impact on prognosis and potentially play a key role in the development and prognosis of oral cancer. Next, the ten candidate genes underwent polygenic overlap through multifactorial Cox regression analysis, and their p-values were calculated using K-M survival analysis. Based on the relative significance of the p-values, the models were ranked, and the model with the most significant p-value was selected as the final prognostic model (Fig. **4C**). Ultimately, a six-gene prognostic model (PFDN1, YARS2, PFKP, ALG2, ANO1, PCMT1) was obtained, with a risk score calculated as follows: risk score = 0.000918 * PFDN1 + 0.004008 * YARS2 + 0.001967 * PFKP + 0.005403 * ALG2 + 0.0004 * ANO1 + 0.00601 * PCMT1. Patients were then divided into high and low-risk groups based on the median risk score. K-M survival analysis results demonstrated that patients in the high-risk group had a shorter overall survival time (Fig. **4D**). Additionally, risk factor plots indicated higher risk scores and more deaths in the high-risk group (Fig. **4E**). Furthermore, principal component analysis (PCA) demonstrated a clear distinction between the high-risk and low-risk groups (Fig. **4F**), thereby substantiating the efficacy of the risk model. To evaluate the predictive efficacy of this risk model, ROC analysis was performed. The AUC values of the model were 0.66, 0.67, 0.63, and 0.7 at the 0.5, 1, 2, 3, and 5-year time points, respectively (Fig. **4G**). These results indicate that the risk model accurately predicts the prognosis of OSCC patients at different time points. Univariate and multivariate Cox analyses demonstrated that the risk model could serve as an independent prognostic indicator for oral cancer patients, irrespective of other clinical factors (Fig. **4H**). Furthermore, concordance index analysis confirmed that the risk model had significantly better predictive efficacy than other clinical indicators. Overall, these findings demonstrate that the risk model performs well and independently predicts the prognosis of oral cancer patients.

### 3.3 External Validation of the Prognostic Model

**Fig. (5):**
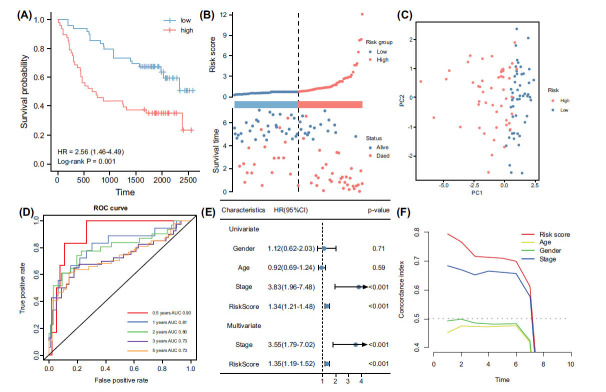
Validation of the risk signature in validation cohort (GEO set). (**A**) The Kaplan-Meier (K-M) algorithm was employed to calculate the difference in survival between the high and low-risk groups in the validation cohort for this risk model. (**B**) The distribution of risk scores and overall survival status was examined for the high- and low-risk groups in the validation cohort. (**C**) The principal component analysis (PCA) was conducted to assess the differentiation between the high and low-risk groups in validation cohort. (**D**) The receiver operating characteristic (ROC) curve was utilized to evaluate the predictive efficacy of the risk model in the validation cohort. (**E**) Univariate and multifactorial COX regression analyses showed that this risk model was also an independent prognostic factor in the validation cohort. (**F**) C-index compares the predictive performance of this risk model with other clinical indicators in the validation cohort.

To assess the stability of the model, we conducted further analyses using an external dataset consisting of 97 OSCC patients. The K-M survival analysis results demonstrated that patients in the high-risk group had a poorer prognosis (Fig. **5A**). Risk factor plots also indicated higher risk scores and a higher number of deaths in the high-risk group (Fig. **5B**). Additionally, PCA analysis revealed a clear differentiation between the high-risk and low-risk groups (Fig. **5C**). Furthermore, the ROC analysis results suggested that the model maintained good predictive efficacy in this validation set, with AUC values of 0.9, 0.81, 0.73, and 0.73 at the 0.5, 1, 2, 3, and 5-year time points, respectively (Fig. **5D**). This indicates that the model remains highly accurate in predicting the prognosis of OSCC patients at different time points. Univariate and multifactorial Cox regression analyses confirmed that the model could serve as an independent prognostic factor for OSCC, influencing patients' prognosis independently of each other and other clinical factors (Fig. **5E**). Moreover, Concordance index analysis results further confirmed that the risk model exhibited better predictive efficacy compared to other clinical indicators (Fig. **5F**). These findings highlight the stable performance and predictive capability of the risk model when validated in an external dataset. The analysis results of the validation set were consistent with those of the training set, providing further support for the model's effectiveness as a powerful tool for the prognostic assessment of OSCC patients.

### 3.4 Prognostic Analysis of the Risk Model in Clinical Subgroups

**Fig. (6):**
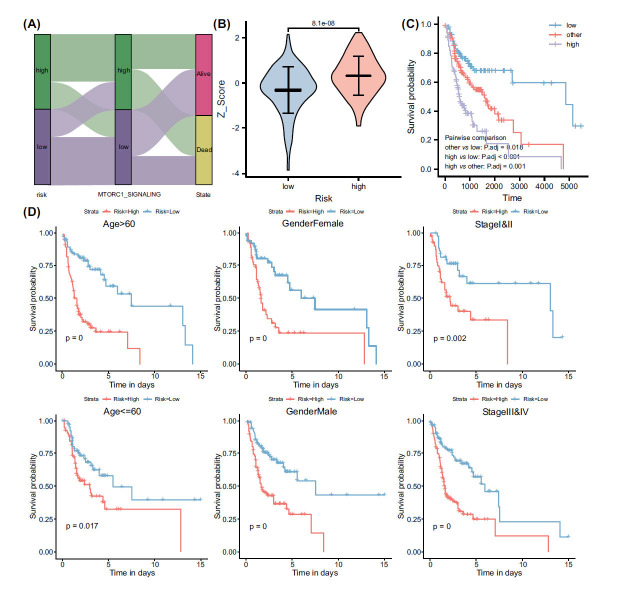
Associations between mTORC1 signaling, risk models, and subgroup analyses of risk models. (**A**) A Sankey diagram illustrating the correspondence between the risk model and mTORC1 signaling, revealing interconnections and dependencies within our dataset. (**B**) Differences in mTORC1 signaling Z-scores between high- and low-risk groups, demonstrating how varying degrees of this signaling pathway's activity can impact a patient's risk classification. (**C**) A two-factor survival analysis of mTORC1 signaling and risk models, displaying the combined effect of these parameters on overall patient survival. (**D**) Kaplan-Meier (KM) survival analysis generating survival curves for various subgroups differentiated by age, gender, and stage, using the risk model as the basis. This plot underscores the prognostic power of the risk model across diverse patient populations. A Mann-Whitney U test (Wilcoxon rank sum test) was used to determine the P values.

Considering that the risk model was derived based on mTROC1 signaling, an alluvial diagram was generated to visually represent the distribution of patients within the high-risk group, revealing a significant overlap with the high-risk mtorc1 group (Fig. **6A**). In addition, a higher mtorc1 z-score was observed in the high-risk group (Fig. **6B**). The results of the two-factor survival analysis demonstrated that patients with both low mTORC1 and low risk exhibited a more favorable prognosis. Conversely, patients belonging to the high mtorc1 and high-risk groups displayed a poorer prognosis (Fig. **6C**). Subsequently, additional analyses were conducted on cases within various clinical subgroups to evaluate the prognostic stratification capability of this risk model. The results demonstrated that the risk model exhibited superior prognostic stratification ability across different age groups, genders, and clinical stages (Fig. **6D**).

### 3.5 Biomarker Expression Validation and Potential Mechanism Analysis

We further confirmed the expression of these six genes within both normal and tumor tissues in our TCGA-HNSC dataset. The results demonstrated that the expression levels of these genes were significantly elevated in tumor tissues compared to normal tissues (Fig. **S1A**). Additionally, we examined the protein expression of these potential biomarkers utilizing the HPA database. The data from immunohistochemistry showed a higher level of expression in tumor tissues, reinforcing our findings (Fig. **S1B**). Following this, we utilized the “limma” package to scrutinize differences in gene expression between high- and low-risk groups. We conducted GO and KEGG enrichment analyses on these divergently expressed genes. The findings suggest a potential involvement of these genes in immune-linked processes, such as the IL-17 signaling pathway and interactions of cytokine receptors (Figs. **S2A** and **2B**). GSEA is a comprehensive approach that interprets gene expression data on a holistic level. This method, in contrast to traditional differential expression analysis, focuses on individual genes, takes into consideration the entirety of the genetic landscape and mitigates issues related to multiple testing. In our study, we employed GSEA to differentiate between high- and low-risk groups. Our results suggested an enrichment of the P53 signaling pathway, cell cycle, and cancer-related pathways within the high-risk group (Fig. **S2C**). Conversely, ether lipid metabolism and primary immunodeficiency were found to be enriched within the low-risk group (Fig. **S2D**).

### 3.6 Correlation Analysis of the Risk Model with Gene Mutations and Immune Microenvironment

**Fig. (7):**
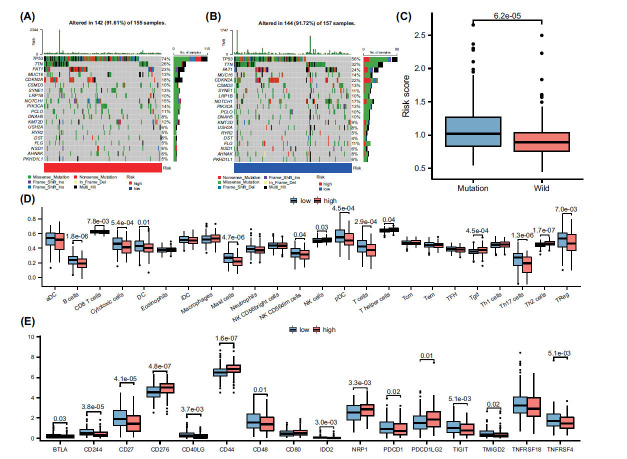
Association of risk models with genetic mutations and the immune microenvironment. (**A**) Depicts the genetic mutations present within the high-risk group of the TCGA cohort, providing insights into the underlying genomic alterations associated with increased disease risk. (**B**) Portrays the gene mutations within the low-risk group of the TCGA cohort, highlighting the distinct mutational landscape characterizing this lower-risk patient population. (**C**) Demonstrates the distribution of risk scores between TP53 mutant and wild-type groups, emphasizing the potential influence of TP53 status on the calculated risk scores. (**D**) Utilizes the single-sample Gene Set Enrichment Analysis (ssGSEA) method to quantify and compare the extent of immune cell infiltration between the high- and low-risk groups in the TCGA cohort, underscoring potential differences in immune response between these groups. (**E**) Compares the expression of immune-related genes between high- and low-risk groups, revealing how immune function may differ according to risk classification. A Mann-Whitney U test (Wilcoxon rank sum test) was used to determine the P values.

Recent studies have highlighted the significance of gene mutations as important factors contributing to poor prognostic survival in patients. To gain further insights into the potential reasons for the prognostic stratification observed in the high and low-risk groups within this risk model, we conducted an analysis of gene mutations among patients in different subgroups. The analysis revealed that the incidence of TP53 gene mutations was higher in the high-risk group (Figs. **7A** and **B**). Additionally, a higher risk score was observed in the TP53 gene mutation group (Fig. **7C**). These findings suggest a correlation between TP53 gene mutations and the increased risk indicated by the risk model. TP53 mutations are known to play a role in cancer development and progression, and their presence may contribute to a poorer prognosis in OSCC patients. Subsequently, to gain insights into the immune microenvironment, we calculated the scores of 24 immune cell types in the TCGA cohort using ssGSEA analysis. The results revealed a higher infiltration of B cells, CD8+ T cells, cytotoxic cells, dendritic cells, mast cells, T cells, and Th17 cells in the low-risk group. Conversely, the high-risk group exhibited higher infiltration of Th2 cells (Fig. **7D**). We further analyzed the expression of immune checkpoint genes across different risk groups. Our results revealed that CD244, TNFRSF4, TMIGD2, CD27, CD48, CD40LG, TIGIT, and PDCD1 were markedly overexpressed in the low-risk group, whereas NRP1, CD276, CD80, CD44, and PDCD1LG2 had notably higher expression in the high-risk group (Fig. **6E**). These findings augment our grasp of the prognostic disparities between high- and low-risk patients within this risk model.

### 3.7 Construction of the Nomogram

**Fig. (8):**
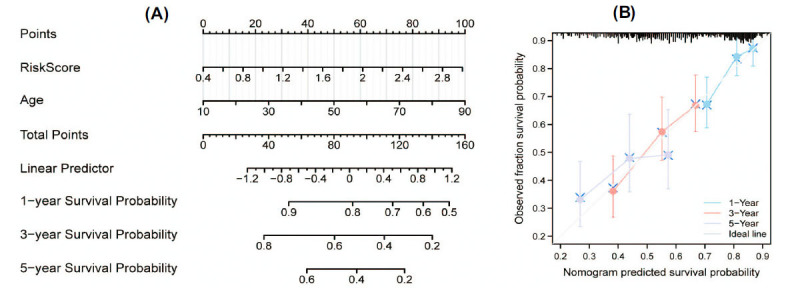
Construction and assessment of the nomogram. (**A**) Demonstrates the creation of a nomogram designed to predict 1-, 3-, and 5-year survival rates in OSCC patients. This model is based on the risk score and age, two critical factors determined to significantly influence patient prognosis. (**B**) Depicts the calibration curve utilized to evaluate the consistency and precision of this nomogram. The close alignment between the predicted and observed outcomes indicates the reliability of the nomogram as a prognostic tool.

To establish a clinically applicable method for predicting survival in patients with OSCC, we identified age and risk models as independent prognostic factors based on the results of previous multifactorial analyses. Utilizing this information, we constructed a Nomogram diagram that combined the risk scores and patient age (Fig. **8A**). Subsequently, we employed a calibration curve to assess the accuracy of the model. The results of the calibration curve demonstrated that the 1-, 3-, and 5-year survival probabilities predicted by the column-line diagram were generally consistent with the actual observed survival probabilities. This validation confirms the reliability and accuracy of the column-line diagram (Fig. **8B**). These findings further emphasize the validity and applicability of our established OSCC survival prediction model. By integrating the risk scores and patient age, we have developed a more precise method for predicting and assessing the survival of OSCC patients. This approach is essential for guiding the development of treatment strategies and improving patient prognosis.

## 4 DISCUSSION

MTORC1 is a protein kinase complex crucial to cell growth regulation and cellular homeostasis [9]. It controls several vital signaling pathways [10] and responds to factors like nutrient availability, energy levels, and environmental stress [11]. Dysregulation of mTORC1 signaling is closely associated with the onset and progression of various diseases. Overactivation of mTORC1 in cancer, for example, fosters abnormal cell proliferation and survival, driving tumor formation and growth [12]. mTORC1 also contributes significantly to diabetes mellitus by regulating essential processes such as the insulin signaling pathway and insulin resistance [13]. Furthermore, abnormalities in mTORC1 signaling have been linked to neurological disorders, including neurodegenerative and mental diseases [14]. Deeper insights into mTORC1 signaling can enhance our understanding of disease mechanisms and guide therapeutic strategy development. Further research will help clarify the exact role of mTORC1 in disease progression, aiding the formulation of targeted treatments.

In this study, we aimed to develop a prognostic model for predicting the survival of OSCC patients based on the mTORC1 signaling pathway, which plays a crucial role in this disease. The constructed 6-gene prognostic model was validated on both training and validation sets, exhibiting robust predictive performance. Comparable studies have surfaced previously, such as Zhang *et al*.'s OSCC prognostic stratification guide based on an epithelial-mesenchymal transition gene prognostic model [15]. Yang *et al*. proposed an OSCC prognosis predicting 17 immune-related gene prognostic models [16], while Huang *et al*. investigated necrotic apoptosis-related genes' role in OSCC, devising a 6-gene prognostic model for overall survival prediction [17]. In contrast, our approach employed the ssGSEA algorithm and Cox proportional hazards regression model to establish the mTORC1 pathway's connection with OSCC patient overall survival. We confirmed the mTORC1 pathway's independence as an OSCC prognostic factor, irrespective of other clinical metrics. Further, by leveraging WGCNA, we located gene modules closely tied to mTORC1 co-expression, which facilitated the discovery of gene clusters strongly connected to the mTORC1 pathway. We ultimately created a six-gene inclusive prognostic model *via* univariate Cox regression, random forests, and multifactorial Cox regression. This model exhibited commendable predictive ability, remarkably demonstrated by AUC values surpassing 0.8 and even reaching 0.9 for 0.5, 1, and 3-year predictions in the validation set. These findings suggest the model's potential as a valuable instrument for more precise OSCC prognosis clinical assessment. To improve the practical applicability of our constructed model within a clinical setting, we integrated the outcomes from the multifactor regression analysis, identifying patient age and risk score as independent prognostic factors. Consequently, we developed a nomogram incorporating these variables to facilitate clinician use. This tool enhances the translation of our prognostic model into routine clinical practice.

TP53 gene mutations are crucial in OSCC development and progression [18]. These mutations can induce malignant behaviors such as abnormal cell proliferation, invasion, and metastasis, impacting patient survival and prognosis. Thus, targeting the TP53 pathway could hinder tumor growth and metastasis, improving OSCC prognosis. Noting significant differences in TP53 mutation rates between high- and low-risk groups, we hypothesize our prognostic model's risk scores may be associated with TP53 mutations. Consequently, future work should focus on incorporating TP53 mutations into the model for better OSCC treatment and monitoring. The tumor immune microenvironment (TIME), composed of various cellular, molecular, and non-cellular elements interacting with tumor cells, plays a key role in tumor development, progression, and prognosis. Studies have linked high infiltration of CD57-expressing natural killer (NK) cells and CD20-positive B cells within the TIME to improved overall and recurrence-free survival [19]. Similarly, our study noted increased NK and B cell infiltration in the low-risk group, possibly due to their roles in eliminating tumor cells. CD57+ NK cells actively kill tumor cells, while CD20+ B cells contribute to the humoral immune response by producing antibodies against pathogens. Thus, boosting NK and B cell presence might control tumor development through an effective immune response. These findings emphasize the importance of understanding OSCC tumor immunology for the development of immunotherapeutic strategies that promote NK and B cell infiltration in the TIME, potentially improving OSCC patient survival and prognosis.

In our study, we pinpointed six potential risk genes associated with OSCC: PFDN1, YARS2, PFKP, ALG2, ANO1, and PCMT1. PFDN1, a co-chaperone protein, primarily captures newly synthesized proteins, subsequently delivering them to the T-complex polypeptide 1, which houses the chaperone protein. Recent investigations have uncovered abnormal overexpression of PFDN1 in various cancers, including colorectal, gastric, lung, and liver. This atypical PFDN1 expression potentially contributes to the uncontrolled proliferation and metastasis of malignant cells, correlating with poor prognosis across diverse tumors [20-23]. In addition, YARS2, a member of mitochondrial tyrosine-tRNA synthetase, exhibits aberrant expression in numerous tumors, correlating with enhanced tumor proliferation and migration [24, 25]. ALG2, a protein capable of binding calcium ions, displays high expression levels in cancerous tissues such as hepatocellular carcinomas, breast carcinomas, lung carcinomas, and gliomas. This abnormal presence is associated with increased tumor cell invasion and drug resistance [26, 27]. PCMT1, a member of the protein repair enzymes, participates in biological processes like apoptosis and tumor metastasis. It has been linked to poor prognosis across several cancers, including liver, bladder, breast, gastric, and prostate cancers [28-31]. Our findings indicate that PFDN1, YARS2, ALG2, and PCMT1 significantly impact OSCC prognosis. Despite these revelations, the precise mechanisms through which these proteins influence OSCC progression warrant further exploration.

Despite the significant findings of our study, we recognize several limitations. Primarily, the use of retrospective data from pre-existing databases may have introduced biases due to factors that were overlooked during the initial data collection or selection processes. Additionally, the retrospective design of our study could result in unmeasured biases and confounders that remain uncontrolled. Although our model produced promising results, further validation through prospective studies is imperative. It is crucial to acknowledge the necessity for both *in vivo* and *in vitro* validation to substantiate our findings under controlled laboratory conditions. These experimental approaches can provide mechanistic insights that are not attainable through clinical data analysis alone. Consequently, future research should prioritize prospective and experimental validation of these results to enhance the depth and robustness of our conclusions.

## CONCLUSION

In summary, our study, employing the ssGSEA, definitively demonstrated the significant impact of the mTORC1 pathway as an autonomous prognostic factor in OSCC. Our findings established a strong association between aberrant activation of the mTORC1 pathway and adverse outcomes in OSCC patients. Utilizing WGCNA and machine learning techniques, we developed and validated a six-gene risk model with independent prognostic significance for OSCC, providing key insights for patients. Additionally, we discovered associations between this risk model and factors such as tumor mutational load and immune-tumor microenvironment infiltration. These findings pave the way for precision medicine implementation and immune-targeted therapeutic approaches in OSCC.

## Data Availability

All data generated or analysed during this study are included in this published article.

## LIST OF ABBREVIATIONS

AUC: area under the curve
Cox-PH: Cox proportional hazards
GEO: Gene Expression Omnibus
GO: Gene Ontology
HNSC: head and neck squamous cell carcinoma
HPA: Human Protein Atlas
K-M: Kaplan-Meier
KEGG: Kyoto Encyclopedia of Genes and Genomes
OS: overall survival
OSCC: Oral squamous cell carcinoma
PCA: principal component analysis
RNA-seq: The RNA sequencing
ROC: receiver operating characteristic
ssGSEA: single-sample gene set enrichment analysis
t-ROC: time-dependent Receiver Operating Characteristic
TCGA: the cancer genome atlas
TIME: tumor immune microenvironment
TPM: transcripts per million mapped reads
WGCNA: Weighted gene co-expression network analysis
