# Supplementary material for: Identification and Verification of a Prognostic Risk Signature in Oral Squamous Cell Carcinoma
Source: Curr Top Med Chem. 2024 Sep 5;25(26):3042–55. doi: 10.2174/0115680266335055240828061128 (PMC12728520; doi:10.2174/0115680266335055240828061128)
Supplement: Supplementary file 1 [file CTMC-25-26-3042_SD1.pdf]

Supplementary Material

Identification and Verification of a Prognostic Risk Signature in Oral Squamous Cell Carcinoma

Rishou Chen[1], Junlin Duan[1], Yonglong Ye[1], Huan Xu[1], Yali Ding[1] and Jun Liu[1,*]

[1]*Laboratory Medicine, Dongguan Hospital of Guangzhou University of Chinese Medicine, Dongguan, China*

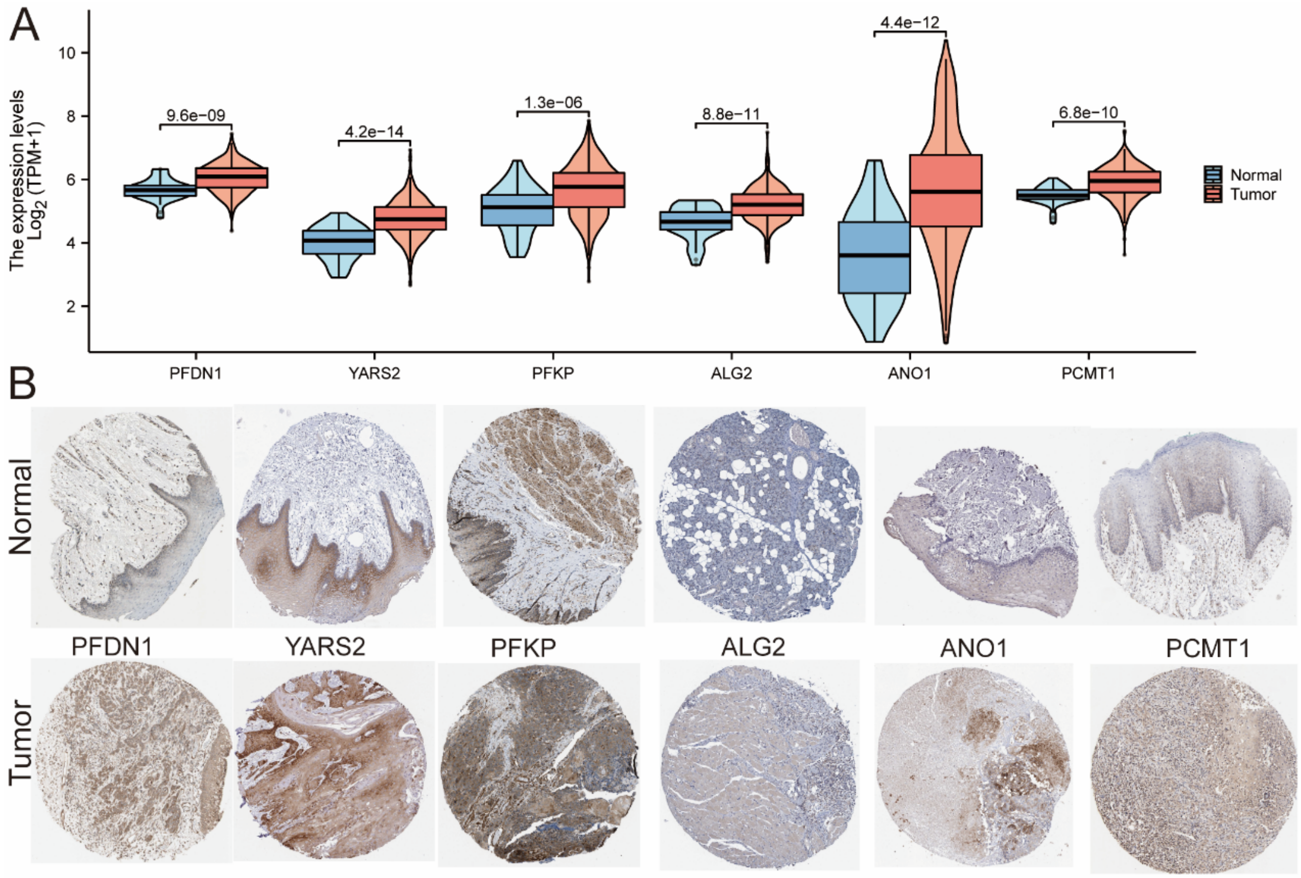

Fig. (S1). Expression validation of potential biomarkers. (**A**) Expression of mRNAs for potential biomarkers in TCGA-HNSC. (**B**) Expression of protein levels of potential biomarkers in HPA data. A Mann-Whitney U test (Wilcoxon rank sum test) was used to determine the P values.

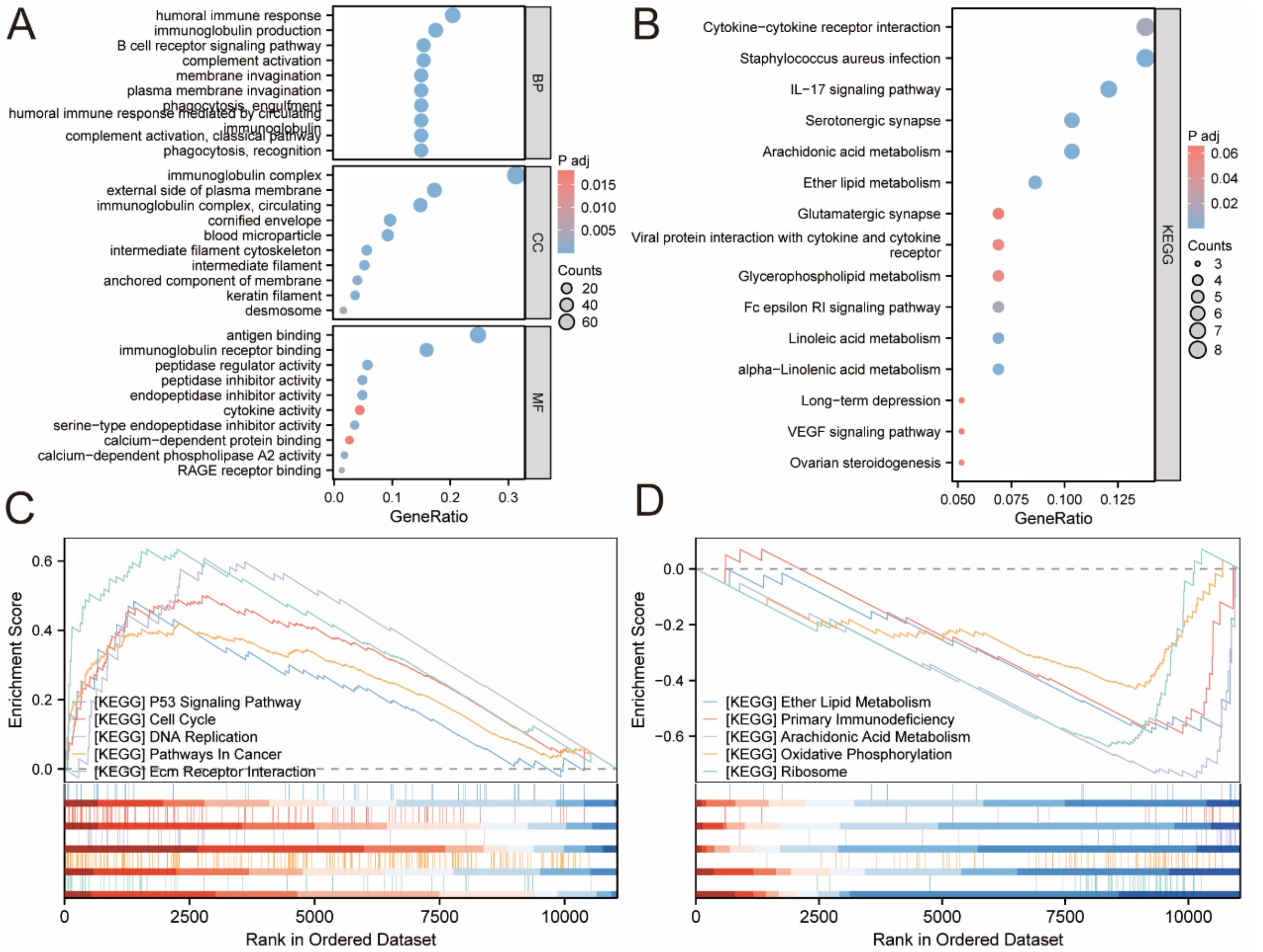

Fig. (S2). Potential Functional Enrichment Analysis. (A) GO and (B) KEGG enrichment analysis of differentially expressed genes in high and low risk groups. GSEA enrichment analysis of the (C) high- and (D) low-risk groups.
